# Calculation of the relative metastabilities of proteins using the CHNOSZ software package

**DOI:** 10.1186/1467-4866-9-10

**Published:** 2008-10-03

**Authors:** Jeffrey M Dick

**Affiliations:** 1 Department of Earth and Planetary Science, University of California, Berkeley, CA 94720, USA

## Abstract

**Background:**

Proteins of various compositions are required by organisms inhabiting different environments. The energetic demands for protein formation are a function of the compositions of proteins as well as geochemical variables including temperature, pressure, oxygen fugacity and pH. The purpose of this study was to explore the dependence of metastable equilibrium states of protein systems on changes in the geochemical variables.

**Results:**

A software package called CHNOSZ implementing the revised Helgeson-Kirkham-Flowers (HKF) equations of state and group additivity for ionized unfolded aqueous proteins was developed. The program can be used to calculate standard molal Gibbs energies and other thermodynamic properties of reactions and to make chemical speciation and predominance diagrams that represent the metastable equilibrium distributions of proteins. The approach takes account of the chemical affinities of reactions in open systems characterized by the chemical potentials of basis species. The thermodynamic database included with the package permits application of the software to mineral and other inorganic systems as well as systems of proteins or other biomolecules.

**Conclusion:**

Metastable equilibrium activity diagrams were generated for model cell-surface proteins from archaea and bacteria adapted to growth in environments that differ in temperature and chemical conditions. The predicted metastable equilibrium distributions of the proteins can be compared with the optimal growth temperatures of the organisms and with geochemical variables. The results suggest that a thermodynamic assessment of protein metastability may be useful for integrating bio- and geochemical observations.

## Background

Owing to the growing body of compositional data for microbial proteins and the exploration of environments that are extreme from the human standpoint, it has become possible in recent years to draw correlations between the compositions of proteins and environmental parameters such as temperature [1]. Accounting for the underlying causes of the observed correlations between environmental parameters and protein composition is an ongoing challenge. Biochemical approaches are based in part on the notion that proteins from thermophilic and hyperthermophilic organisms should have greater structural stabilities than their mesophilic counterparts [2]. Compositional features of thermophilic proteins that may enhance their structural stabilities include increased numbers of hydrophobic residues, stronger charge interactions on the protein surfaces, and other properties of the amino acid sequence [3]. However, it has also been suggested that, at least for sulfur, the elemental makeup of proteins is correlated with the chemical compositions of the environment [4]. This study was motivated by the desire to explore a possible thermodynamic explanation for the relationship between protein composition and the extracellular environment, which is shaped in part by geochemical constraints.

A thermodynamic assessment of protein metastability provides a framework for describing the relationship between geochemistry and protein composition that until now has received relatively little attention. The geochemical literature abounds with examples of theoretical calculation of the compositions of stable and/or metastable equilibrium reference states as a way to predict the distributions of, and reaction pathways among, minerals and inorganic or organic aqueous species [5,6]. In recent years, the calculation [7-11] and experimental investigation [12-14] of metastable equilibrium states in biogeochemical systems has gained traction. The primary advantage of extending a framework of this type to proteins and other biomacromolecules is that it places biochemical reactions in the same context as observations on the inorganic systems to which microbial metabolic pathways are coupled. Temperature, pressure, oxidation state and pH are just some of the variables that are commonly measured in geochemical studies that also appear explicitly in the thermodynamic representation of protein metastability reactions.

This study was undertaken in order to explore the thermodynamic relationships between geochemical variables and protein composition for model proteins from a number of organisms adapted to different environments. The cell-surface glycoproteins in archaea and the surface-layer proteins in bacteria [15,16] were chosen for this purpose because they are intimately associated with the extracellular aquatic and mineralogical setting.

Because experimental values of the standard molal Gibbs energies of the model proteins were not available, they were calculated using previously reported group additivity and equations of state algorithms that are referenced to ionized unfolded aqueous proteins [17,18]. These values are requisite for calculating the composition of the metastable equilibrium state in an open system described by chemical potentials of basis species, or perfectly mobile components [19-22]. The predicted chemical activities of species can then be displayed on chemical predominance and/or speciation diagrams whose axes correspond to intensive chemical variables. Because of the lack of integration of algorithms for calculating thermodynamic properties of proteins in available geochemical equilibrium software packages, the task of calculating and graphically representing the metastable equilibrium distributions of the proteins was managed through development of the CHNOSZ software package, which is introduced in this study.

The implementation of the thermodynamic algorithms and data into the package is described first below. The results of the calculations for the model system of proteins are then described and are displayed primarily in the form of diagrams depicting the calculated metastable equilibrium distributions of the proteins. The graphical depictions shown below are only limited portrayals of the metastable equilibrium states of systems of proteins, which are in fact multidimensional functions of thermodynamic variables. The predicted response of at least one of the metastability reactions between proteins from hyperthermophilic and mesophilic organisms appears to be aligned with the differences in temperature, pressure and oxidation state between their environments. However, more tests in other systems will be required to assess the generality of the approach. Some potential implications of the findings are addressed briefly in the concluding remarks, and the paper is finished with a section devoted to the methods adopted for writing protein metastability reactions and computing their thermodynamic properties.

## Implementation

The CHNOSZ software package consists of source code, data files, and documentation. It is written for the cross-platform R software environment [23]. The package can be freely downloaded from the project website at . The features of the package, its basic program structure, and the thermodynamic database are summarized in the following paragraphs.

### Features

CHNOSZ was developed in order to ease calculations of 1) the standard molal thermodynamic properties of chemical species and reactions as a function of temperature and pressure, 2) the standard molal thermodynamic properties and equations of state parameters of neutral and ionized proteins using group additivity algorithms, 3) the chemical affinities of formation reactions of species of interest from basis species describing the system, and to assist in 4) generating metastable equilibrium activity diagrams for systems of biomolecules and/or other species.

The functions provided in CHNOSZ are suitable for either interactive use or scripted operation. The diagrams that are produced can be viewed on screen or saved as postscript files. Because the thermodynamic database includes the chemical formulas of species in addition to their standard molal thermodynamic properties, functions operating on user-input chemical reactions have the option to check, and possibly automatically correct, the mass balance of the reactions. This feature can speed up user interaction with the program and the writing of program scripts. The program has been designed with features in mind and is not presently optimized for speed. Most of the diagrams shown below can be produced in under a minute, but temperature-pressure diagrams of the same resolution require substantially more computational time, owing to the number of times the equations of state subroutines are called.

The package was developed with the goal of analyzing protein reactions, but the range of systems that can be studied using the software is limited only by the species available in the thermodynamic database, to which the user can make either temporary or persistent additions or updates. Complete documentation of the functions, including examples derived from the geochemical literature and this study, is provided with the package. Usage of the major functions in CHNOSZ is summarized below.

### Standard molal properties

**Figure 1:**
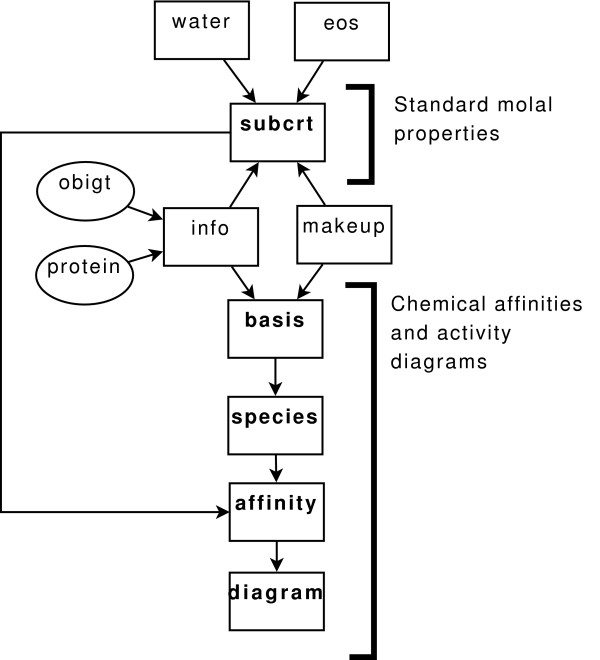
**Functions and data flow in the CHNOSZ program**. Data sources are represented by ellipses, and functions by boxes. Computations in CHNOSZ are initiated by the user accessing the primary functions, shown in bold font. The accessory functions, shown in normal font, perform many of the underlying calculations.

The relationships among the primary functions provided in CHNOSZ and some of the accessory functions are depicted in the flowchart shown in Fig. 1. Calculation of the standard molal thermodynamic properties of species and chemical reactions as a function of temperature and pressure is implemented in the primary function subcrt. The name of this function is a variation of the name of the SUPCRT92 software package [24]. The temperature and pressure ranges of calculations possible using subcrt are the same as those for SUPCRT92.

The accessory function water implements two computational options for calculating the thermodynamic and electrostatic properties of liquid H_2_O as a function of temperature and pressure. The first of these options provides an interface to the FORTRAN subroutine named H2O92D.F that was distributed with SUCPRT92 [24] and that is included in the CHNOSZ source package. The calculation of the properties of liquid H_2_O in this case is consistent with data and equations from Refs. [25-27] and others (see Ref. [24]). The stated temperature and pressure limits of applicability for these calculations, described in Ref. [24], are from 0.01°C and *P*_SAT _(*i.e*., 1 bar at temperatures below 100°C and the saturation vapor pressure of H_2_O at higher temperatures) to 2250°C and 30000 bar. However, electrostatic properties of the solvent, which are required by the revised Helgeson-Kirkham-Flowers (HKF) equations of state for aqueous species, can not be computed above 1000°C and 5000 bar. An alternative computational option for the properties of liquid H_2_O corresponds to the IAPWS-95 formulation for thermodynamic properties [28] coupled with equations for electrostatic properties taken from Ref. [29].

The functions denoted by eos in Fig. 1 actually consist of two functions, hkf, for calculating as a function of temperature and pressure the standard molal thermodynamic properties of aqueous species using the revised HKF equations of state [30-33], and cgl, for calculating the properties of crystalline, gaseous and liquid (except H_2_O) species. The heat capacity equation implemented in CHNOSZ for these species contains up to six terms, as used in Ref. [34]; the first three terms are those in the Maier-Kelley equation [35,36] which is used in the SUPCRT92 package.

The accessory function info provides a bridge between the thermodynamic and protein databases and the other functions. The function known as makeup is concerned with conversion between various computer- and human-readable representations of the chemical compositions of species. Its primary purpose is to transform the chemical formulas of species contained in the thermodynamic database (*e.g*., 'C4H6NO4-' for aspartate) into dataframe objects (which in R are similar to matrices with named columns and rows) so that other functions or makeup itself can perform further calculations on the stoichiometries of species. This function is also responsible for transforming a compositional dataframe back into a one-line chemical formula, and for calculating the reaction coefficients of basis species in formation reactions of the species of interest. It is with the aid of this function that subcrt checks whether a user-input chemical reaction is balanced with respect to mass and charge and automatically corrects the reaction if the necessary basis species have been defined.

**Figure 2:**
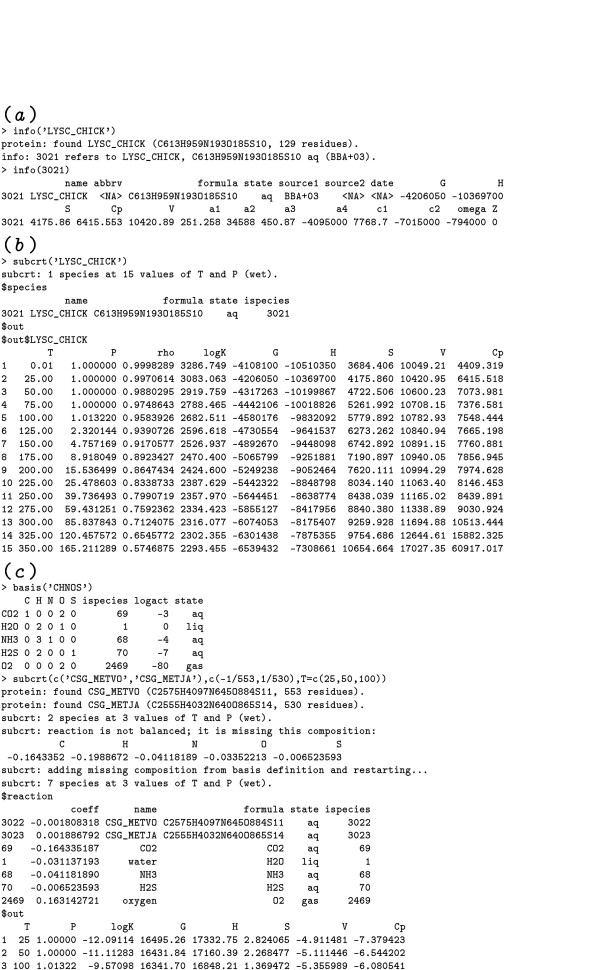
**Transcript of CHNOSZ session to calculate thermodynamic properties of proteins and reactions**. Commands at the prompt (>) were entered to calculate (*a*) the standard molal thermodynamic properties at 25°C and 1 bar and equations of state parameters of nonionized chicken lysozyme (LYSC_CHICK), (*b*) the standard molal thermodynamic properties of lysozyme as a function of temperature at *P*_SAT _and (*c*) the standard molal properties of the nonionized counterpart to Reaction 1 as a function of temperature at *P*_SAT_.

Examples of the usage of the info and subcrt functions are shown in the program transcript in Fig. 2. The standard molal thermodynamic properties at 25°C and 1 bar and the equations of state parameters of chicken lysozyme (LYSC_CHICK, accession no. P00698 in the Swiss-Prot database [37]) can be retrieved using the code shown in Fig. 2a. The properties and parameters whose values appear in the example are standard molal Gibbs energy (Δ*G*°) and enthalpy (Δ*H*°) of formation from the elements (cal mol^-1^), standard molal entropy (*S*°), heat capacity ($C_{P}^{\circ }$) and *c*_1 _(cal K^-1 ^mol^-1^), standard molar volume (*V*°) (cm^3 ^mol^-1^), *a*_1 _(cal bar^-1 ^mol^-1^), *a*_2 _and *ω *(cal mol^-1^), *a*_3 _(cal K bar^-1 ^mol^-1^), and *a*_4 _and *c*_2 _(cal K mol^-1^). The parameters *a*_1_, *a*_2_, *a*_3_, *a*_4_, *c*_1_, *c*_2 _and *ω *are species-dependent coefficients in the revised HKF equations of state. Note that the properties and parameters of proteins returned by info are those of nonionized proteins; the ionization contributions to thermodynamic properties of proteins are calculated using a separate function. Sample code for calculating the standard molal thermodynamic properties of LYSC_CHICK as a function of temperature at *P*_SAT _is shown in Fig. 2b, where the units are °C (*T*), bar (*P*), g cm^-3 ^(*ρ*, density of water) and those listed above for the standard molal properties. The reaction-balancing feature of subcrt is demonstrated in Fig. 2c for Reaction 1 (below). In this mode, all the user has to do is identify the basis species in the system and the reaction coefficients of the proteins, and the program finds the correct quantities of basis species to add to the reaction.

### Chemical affinities and metastability diagrams

The primary function subcrt and the related accessory functions permit calculation of the standard molal Gibbs energies of protein formation reactions and corresponding values of the equilibrium constants (*K*_*r *_in Eqn. M7). Calculation of the activity products and chemical affinities of reactions (*Q*_*r *_and ***A***_*r *_in Eqn. M7) is implemented in the sequence of primary functions basis, species, affinity that is depicted in Fig. 1.

Two conditions are required of a valid set of basis species in CHNOSZ: 1) the number of basis species is equal to the number of elements (and charge, if present). 2) The stoichiometric matrix denoting the elemental composition (and charge if present) of the basis species, which is square according to condition (1), is non-singular and has a real inverse. These two conditions ensure that a formation reaction for any species of interest in the system can be written using only positive or negative real numbers as reaction coefficients on the basis species. The basis species themselves can be any species that are present in the thermodynamic database, including nonionized proteins. The function basis also permits redefining the physical states of basis species (if a corresponding species in that state is present in the thermodynamic database) and/or setting the activities (*a*) or fugacities (*f*) of the basis species to be used in the following calculations. These values have default settings given by log *a *= -3 for aqueous species, log *f *= 0 for gases and log *a *= 0 for other species. The function basis can also be used to assign a buffer to one or more basis species so that the activities or fugacities of those basis species are taken from the buffer system.

After defining the basis species, the user can select any number of species of interest using the primary function species. The user may also call species to remove species or to alter the chemical activities or fugacities of the species of interest to be used in the calculations of chemical affinity. These values default to log *a *= -3 for aqueous species, log *f *= 0 for gases and log *a *= 0 for other species.

The function affinity permits calculation of log *Q*_*r *_and ***A***_*r *_of formation reactions (such as those represented generically by Reaction M1) using Eqn. (M7) taking into account the activities and/or fugacities of the basis species and the species of interest. The contributions of the *Q*_*r *_and *K*_*r *_terms to the calculation are denoted conceptually in Fig. 1 by the two arrows, from the top and left, respectively, pointing toward the box labeled affinity. The calculations of chemical affinity can be carried out at a single point in temperature, pressure, chemical activity space, or as a function of one or two of *T*, *P *and logarithms of chemical activity or fugacity of the basis species. The accessory function buffer is invoked by affinity if one or more basis species were previously associated with a buffer system; the activities or fugacities of the basis species constrained in this way are then used by the program to calculate log *Q*_*r *_using Eqn. (M5).

The results of the calculations performed by affinity are accepted as input by diagram, which produces the diagrams using plotting functions provided in the R distribution. Many options are available for adding labels and legends and otherwise customizing the plot style.

### Thermodynamic database

The database of thermodynamic properties packaged with CHNOSZ is contained in a file named OBIGT.csv. Work on this database was motivated by a software project developed by H. C. Helgeson and coworkers, named OrganoBioGeoTherm, that provides a Windows interface to the SUPCRT92 program (J. J. Donovan, personal communication).

The thermodynamic data file has records for over 2500 inorganic, organic and biochemical crystalline, gaseous, liquid and aqueous species. The thermodynamic data were originally taken from the data file distributed with the SUPCRT92 package. Updates since that time were taken from the SLOP98 data file downloaded from  and from recent reports of thermodynamic data and revised HKF equations of state parameters for aqueous inorganic and organic species, as well as proteins and other species of biogeochemical interest [[38-40], and others]. The records in the data file include the names, states and chemical formulas of the species, up to two literature citations, and values of the standard molal thermodynamic properties at 25°C and 1 bar and equations of state parameters. The comma-separated-value (.csv) file format permits rapid reading of the data file by the CHNOSZ program or other software as well as addition to or modification of the file contents by the user. The CHNOSZ package also provides utility functions that can be used to export or import thermodynamic data to or from the SUPCRT92 data file format.

The data file protein.csv of amino acid compositions of proteins has records for over 200 proteins including those referred to in the present study. The user can add the composition of a protein to CHNOSZ by modifying this file, or at run time by inputting the amino acid composition of the protein at the command line or requesting a search of the online Swiss-Prot database [37] through the function called protein.

## Results

The model cell-surface proteins used in this study are listed in Table 1. The selected organisms were chosen to represent diverse geochemical environments. It can be seen from the optimal growth temperatures given in Table 1 that three of the organisms (*M. jannaschii*, *M. sociabilis *and *M. fervidus*) are hyperthermophilic, others such as *M. voltae *are mesophilic, and one organism (*M. burtonii*) is psychrotolerant. The chemical formulas and standard molal Gibbs energies of the proteins shown in Table 1 are those calculated for the nonionized aqueous proteins. Although the real proteins form crystalline or paracrystalline lattices on the cell surface [41], we are restricted at this time to using an aqueous group additivity model for lack of a crystalline analog. The present formulation is also restricted to the polypeptide molecules of proteins and does not take account of the presence of the carbohydrate chains in the glycoproteins. The standard molal Gibbs energies of ionized proteins were calculated in the present study by combining those of the nonionized proteins with ionization contributions (see Ref. [18] and the Methods).

**Table 1:** Model proteins used in the present study.

Organism	*T*_opt_^**a**^	Protein^**b**^	ID	Length	Formula^**c**^	$\Delta G_{f}^{\circ c}$
*Methanothermus sociabilis*	88 [62]	CSG_METSC	P27374	571	C_2812_H_4405_N_747_O_872_S_16_	-21875
*Methanocaldococcus jannaschii*	85 [43]	CSG_METJA	Q58232	530	C_2555_H_4032_N_640_O_865_S_14_	-24236
*Methanothermus fervidus*	83 [63]	CSG_METFE	P27373	571	C_2815_H_4411_N_747_O_872_S_14_	-21796
*Haloarcula japonica*	42 [64]	CSG_HALJP	Q9C4B4	828	C_3669_H_5647_N_971_O_1488_	-43458
*Methanococcus voltae*	38 [42]	CSG_METVO	Q50833	553	C_2575_H_4097_N_645_O_884_S_11_	-24881
*Methanococcoides burtonii*	23 [65]	CSG_METBU	Q12YZ7	278	C_1362_H_2111_N_355_O_442_S_4_	-11677
*Acetogenium kivui*	66 [66]	SLAP_ACEKI	P22258	736	C_3584_H_5648_N_926_O_1138_S	-29331
*Bacillus stearothermophilus*	65 [67]	SLAP_BACST	P35825	1198	C_5676_H_9113_N_1489_O_1863_S_3_	-48792
*Bacillus licheniformis*	50 [68]	SLAP_BACLI	P49052	844	C_3977_H_6396_N_1068_O_1286_S_2_	-33598
*Aeromonas salmonicida*	23 [69]	SLAP_AERSA	P35823	481	C_2250_H_3580_N_618_O_716_S_2_	-18233

**a**. *T*_opt _stands for laboratory optimal growth temperatures in °C, which were taken from the indicated sources. **b**. Compositions of cell-surface glycoproteins (CSG) from archaea and surface-layer proteins (SLAP) from bacteria were taken from the Swiss-Prot/UniProt database [37] using the noted accession numbers (IDs). Signal sequences were excluded where annotated, and names of the proteins except CSG_METBU correspond to those used in the Swiss-Prot database. **c**. Chemical formulas and standard molal Gibbs energies of formation from the elements at 25°C and 1 bar ($\Delta G_{f}^{\circ }$, in kcal mol^-1^) were calculated for nonionized aqueous proteins.

The relative metastabilities of the model proteins were calculated as a function of temperature, pressure and chemical activities or fugacities of basis species. Results of the calculations are presented below primarily on metastable equilibrium activity diagrams depicting either the predominant protein species as a function of two intensive variables, or on speciation diagrams showing the metastable equilibrium chemical activities of proteins as a function of a single variable. The computations were carried out using the CHNOSZ software package together with a program script for use with the package that is provided in Additional File 1.

### Predominance diagrams

To assess the relative metastabilities of surface-layer proteins from different organisms as a function of temperature, pressure and oxidation state, we can first write a reaction between the cell-surface proteins from *M. voltae *and *M. jannaschii *as

(1) $$\begin{array}{c} \frac{1}{553}{\text{C}}_{2575}{\text{H}}_{4040.935}{\text{N}}_{645}{\text{O}}_{884}{\text{S}}_{11_{\left(\text{CSG\_METVO},aq\right)}}^{-56.065}+0.164{\text{CO}}_{2(aq)}+0.031{\text{H}}_{2}\text{O}+0.041{\text{NH}}_{3(aq)}+0.006{\text{H}}_{2}{\text{S}}_{\left(aq\right)}\\ ⇌\frac{1}{530}{\text{C}}_{2555}{\text{H}}_{3976.130}{\text{N}}_{640}{\text{O}}_{865}{\text{S}}_{14_{\left(\text{CSG\_METJA},aq\right)}}^{-55.870}+0.163{\text{O}}_{2(g)}+0.004{\text{H}}^{+}, \end{array}$$

which is a specific statement of Reaction M2 for the ionized proteins. The coefficient in front of each of the protein formulas is the reciprocal of the number of amino acid residues in the corresponding protein. Hence, protein length is conserved in Reaction 1. Let us now write a specific statement of Eqn. (M8) for Reaction 1 as

(2) $$\log K_{1}=A_{1}/2.303RT+\log\frac{a_{\text{CSG\_METJA}}^{1/530}}{a_{\text{CSG\_METVO}}^{1/553}}+\log\frac{f_{{\text{O}}_{2(g)}}^{0.163}a_{\text{H}+}^{0.004}}{a_{{\text{CO}}_{2(aq)}}^{0.164}a_{{\text{H}}_{2}\text{O}}^{0.031}a_{{\text{NH}}_{3(aq)}}^{0.041}a_{{\text{H}}_{2}\text{S}}^{0.006}},$$

where *R *stands for the gas constant and log *K*_1 _and ***A***_1 _denote, respectively, the logarithm of the equilibrium constant and the chemical affinity of Reaction 1.

**Figure 3:**
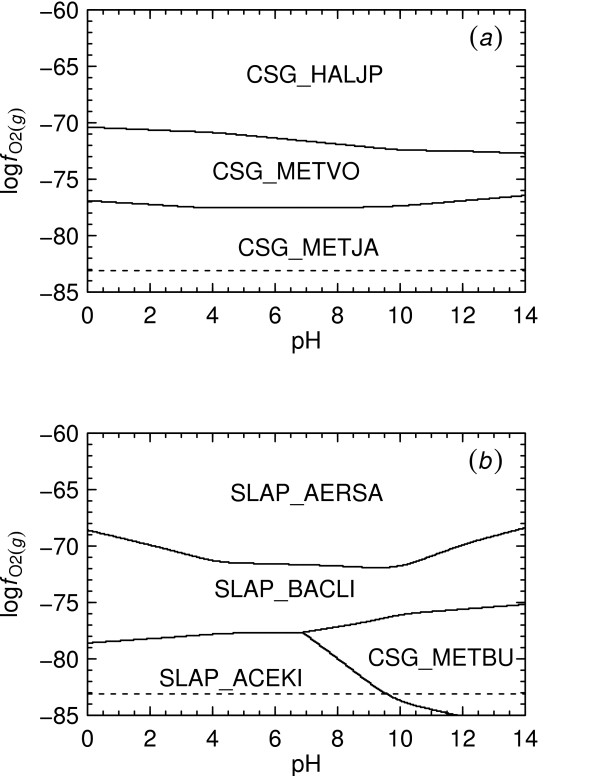
**Relative metastabilities of proteins**. log $f_{{\text{O}}_{2(g)}}$-pH diagrams at 25°C and 1 bar were constructed using activities of the basis species given in the Methods. Predominance field boundaries correspond to metastable equilibrium activities of proteins equal to 10^-3^. The diagrams were made for (*a*) all of the proteins listed in Table 1 and (*b*) the proteins listed in Table 1 except for those appearing in the first diagram. The dashed line appearing in each diagram represents the lower (reducing) stability limit of H_2_O.

The equal-activity boundary shown in Fig. 3a between CSG_METVO and CSG_METJA is consistent with metastable equilibrium between the proteins, or ***A***_1 _= 0. The location of the boundary can be calculated by combining Eqn. (2) with ***A***_1 _= 0, the equilibrium constant of the reaction, and the reference activities of the basis species and proteins. In this study, the reference activities of the proteins were set to 10^-3 ^and those of the basis species set to the values listed in the Methods.

**Figure 4:**
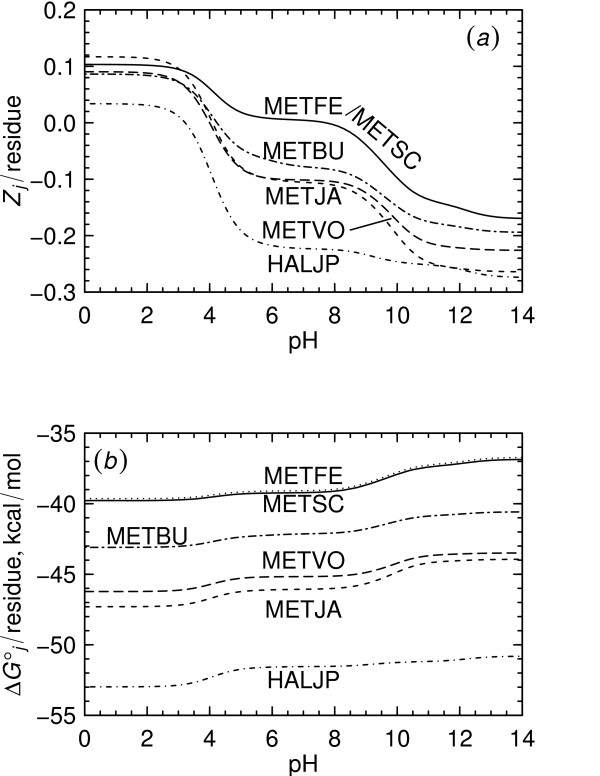
**Properties of archaeal surface-layer proteins**. Shown are calculated values of the net charge per residue (*a*) and standard molal Gibbs energy of formation from the elements (*b*) at 25°C and 1 bar for surface-layer proteins from archaeal species listed in Table 1. The computed charges per residue of CSG_METFE and CSG_METSC are indistinguishable from one another in (*a*).

In Reaction 1 it can be noted that O_2(*g*) _appears on the same side of the reaction as ${\text{C}}_{2555}{\text{H}}_{3976.130}{\text{N}}_{640}{\text{O}}_{865}{\text{S}}_{14_{\left(\text{CSG\_METJA},aq\right)}}^{-55.870}$; hence, the metastability of this protein is increased relative to that of ${\text{C}}_{2575}{\text{H}}_{4040.935}{\text{N}}_{645}{\text{O}}_{884}{\text{S}}_{11_{\left(\text{CSG\_METVO},aq\right)}}^{-56.065}$ by decreasing log $f_{{\text{O}}_{2(g)}}$, which can be seen in Fig. 3a. It is also apparent from Fig. 3a that at pH 7, the formation of CSG_METJA is predicted to be favored by increasing pH. However, at pHs less than ~6, increasing pH favors formation of CSG_METVO. This observation is consistent with the variation in the charges of the proteins as a function of pH, which are shown normalized to the lengths of the proteins in Fig. 4a. For example, at pH 2, the charge per residue of CSG_METJA is greater than that of CSG_METVO, and a statement of Reaction 1 written for the proteins in their calculated ionization states at this pH would have H^+ ^as a reactant instead of a product. The standard molal Gibbs energies of the ionized proteins which were used to calculate log *K*_1 _are depicted in Fig. 4b per residue of protein.

Figure 3a was generated in CHNOSZ using a sequence of commands similar to the following. The complete program script for this and the other figures is provided in Additional File 1:

(3) $$\begin{array}{l} \text{basis}("\text{CHNOS}+")\\ \text{species}(\text{c}("\text{CSG\_METSC}","\text{CSG\_METJA}",\text{CSG\_METFE}",\\ "\text{CSG\_HALJP}","\text{CSG\_METVO}","\text{CSG\_METBU}","\text{SLAP\_ACEKI}",\\ "\text{SLAP\_BACST}","\text{SLAP\_BACLI}","\text{SLAP\_AERSA}"))\\ \text{a}<-\text{affinity }(\text{pH}=\text{c }(0,14),\text{ O}2=\text{c }(-85,-60))\\ \text{diagram}(\text{a}) \end{array}$$

Execution of the first command shown in Example 3 defines the basis species characterizing the chemical system. Here, 'CHNOS+' is a keyword that identifies the basis species used in this paper and that appear in Reaction 1. The second command defines the species of interest, corresponding to the proteins listed in Table 1. With the third command, the chemical affinities of the formation reactions of each of the proteins are calculated on a two-dimensional grid as a function of pH and log $f_{{\text{O}}_{2(g)}}$ and the results assigned to a temporary object. Finally, the fourth command instructs the program to produce a metastable equilibrium activity diagram for the system, which in this case is a predominance diagram as a function of pH and log $f_{{\text{O}}_{2(g)}}$. The reference temperature and pressure and activities of the basis species and proteins are not explicitly specified in Example 3, and are set to default values by the program that correspond to those described in the Methods.

The approach used in CHNOSZ to make predominance diagrams does not rely on writing metastability reactions as represented by Reaction 1 but instead on using formation reactions for the proteins. For example, a specific statement of Reaction M1 for CSG_METJA in its computed ionization state at 25°C, 1 bar and pH 7 is

(4) $$\begin{array}{l} 2555{\text{CO}}_{2(aq)}+1042{\text{H}}_{2}\text{O}+640{\text{NH}}_{3(aq)}+14{\text{H}}_{2}{\text{S}}_{\left(aq\right)}\\ \;⇌{\text{C}}_{\text{2555}}{\text{H}}_{3976.130}{\text{N}}_{640}{\text{O}}_{865}{\text{S}}_{14_{\left(\text{CSG\_METJA},aq\right)}}^{-55.870}+2643.5{\text{O}}_{2(g)}+55.870{\text{H}}^{+}. \end{array}$$

Using CHNOSZ, the chemical affinities of Reaction 4 and its counterparts for any other specified proteins of interest are first computed using Eqn. (M7). The chemical affinities of the formation reactions are then compared with one another to determine the theoretically predominant protein given the input conditions, which is the one with the highest chemical affinity of formation per residue. In this way, it is possible to generate predominance diagrams like those shown in Figs. 3a and 3b for any number of proteins. The diagram shown in Fig. 3a was produced using all ten proteins listed in Table 1, but only some of the proteins predominate at different points in the diagram. Removing these proteins from consideration leads to the results shown in Fig. 3b, where the metastability relationships among some of the less metastable proteins are depicted.

### Chemical activity (speciation) diagrams

**Figure 5:**
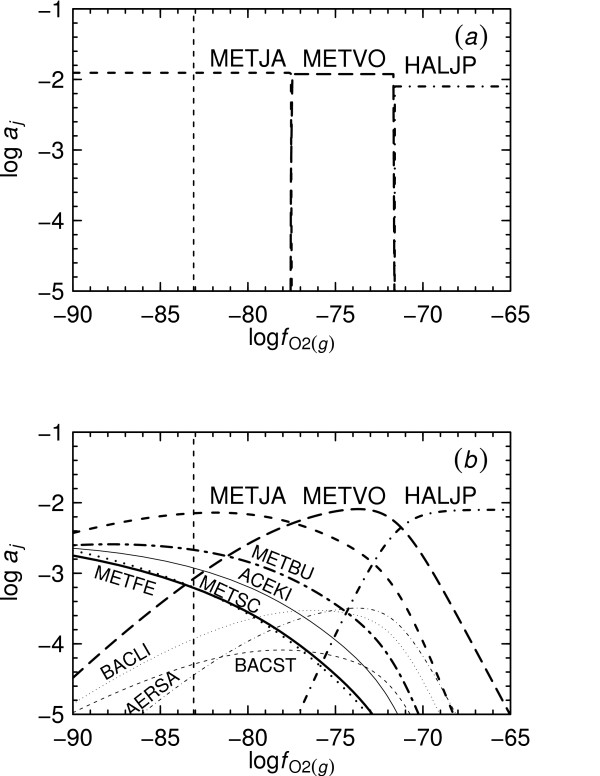
**Metastable equilibrium chemical activities of proteins**. Logarithms of chemical activities of the proteins listed in Table 1 were calculated at 25°C and 1 bar using reactions written for (*a*) whole protein formulas or (*b*) residue equivalents of the proteins. Activities of the basis species were set to the values given in the Methods, and initial activities of the proteins were set to 10^-3^. The vertical dashed lines represent the lower stability limit of H_2_O.

To calculate the chemical activities of proteins in metastable equilibrium, let us consider two ways of writing the formulas of proteins in chemical reactions. The first is represented in Reaction 1 above, in which are entered the whole formulas of proteins. If the conditions are such that metastable equilibrium between the proteins in this reaction corresponds to activities of the proteins each equal to 10^-3^, we have in Eqn. (2) $\log\left(a_{\text{CSG\_METJA}}^{1/530}/a_{\text{CSG\_METVO}}^{1/553}\right)$ = -0.0002. If we decrease log $f_{{\text{O}}_{2(g)}}$ by a single unit, it follows from Eqn. (2) that $\log\left(a_{\text{CSG\_METJA}}^{1/530}/a_{\text{CSG\_METVO}}^{1/553}\right)$ = -0.0002 + 0.163 = 0.1628. Accordingly, supposing that *a*_CSG_METJA _is held constant at 10^-3^, the activity of CSG_METVO would be ~10^-93^, a vanishingly small quantity. The relative metastabilities of proteins computed using this approach are shown graphically in Fig. 5a, where it can be seen that the logarithms of activities of the non-predominant proteins drop precipitously.

Let us propose to write the formulas of proteins in metastability reactions as residue equivalents instead of whole protein formulas. The chemical formula or any standard molal thermodynamic property of a residue equivalent of a protein is defined to be that of the protein divided by the length of the protein. In contrast, assuming activity coefficients of proteins and residue equivalents to be unity, the chemical activity of the residue equivalent of the *j*th protein (*a*_residue, *j*_) is equal to the chemical activity of the protein (*a*_*j*_) multiplied by the length of the protein (*n*_*j*_):

(5) *a*_residue, *j *_= *n*_*j *_× *a*_*j*_.

We can rewrite Reaction 1 in terms of the residue equivalents of the proteins as

(6) $$\begin{array}{c} {\text{C}}_{4.656}{\text{H}}_{7.307}{\text{N}}_{1.166}{\text{O}}_{1.598}{\text{S}}_{0.020_{\left(\text{residue},\text{CSG\_METVO},aq\right)}}^{-0.101}+0.164{\text{CO}}_{2(aq)}+0.031{\text{H}}_{2}\text{O}+0.041{\text{NH}}_{3(aq)}+0.006{\text{H}}_{2}{\text{S}}_{\left(aq\right)}\\ ⇌{\text{C}}_{4.821}{\text{H}}_{7.502}{\text{N}}_{1.208}{\text{O}}_{1.632}{\text{S}}_{0.026_{\left(\text{residue},\text{CSG\_METJA},aq\right)}}^{-0.105}+0.163{\text{O}}_{2(g)}+0.004{\text{H}}^{+}. \end{array}$$

In Reaction 6, the coefficients on the reactant and product residue equivalents are both set to unity. Hence, in both Reactions 1 and 6 protein length is conserved. Using Eqn. (M8) we can write for Reaction 6,

(7) $$\log K_{6}=A_{6}/2.303RT+\log\frac{a_{\text{residue},\text{CSG\_METJA}}}{a_{\text{residue},\text{CSG\_METVO}}}+\log\frac{f_{{\text{O}}_{2(g)}}^{0.163}a_{\text{H}+}^{0.004}}{a_{{\text{CO}}_{2(aq)}}^{0.164}a_{{\text{H}}_{2}\text{O}}^{0.031}a_{{\text{NH}}_{3(aq)}}^{0.041}a_{{\text{H}}_{2}\text{S}}^{0.006}}.$$

Let us now consider conditions such that the metastable equilibrium activities of the proteins are each equal to 10^-3^. From Eqn. (5) we have *a*_residue, CSG_METJA _= 0.530 and *a*_residue,CSG_METVO _= 0.553, so log (*a*_residue,CSG_METJA_/*a*_residue,CSG_METVO_) = - 0.018. Now, if log $f_{{\text{O}}_{2(g)}}$ is decreased by one unit, it follows from Eqn. (7) that to maintain metastable equilibrium, log (*a*_residue,CSG_METJA_/*a*_residue,CSG_METVO_) = -0.018 + 0.163 = 0.145. Supposing *a*_residue,CSG_METJA _to be held constant at 0.530 (*a*_CSG_METJA _= 10^-3^), *a*_residue,CSG_METVO _would be 0.380 (*a*_CSG_METVO _= 10^-3.16^). This type of assessment leads to the results shown graphically in Fig. 5b, where it can be seen that the metastable equilibrium activities of the proteins as a function of log $f_{{\text{O}}_{2(g)}}$ are within a few log units of each other, even for the non-predominant proteins.

The diagram shown in Fig. 5b was actually constructed using CHNOSZ by taking account of the formation reactions of residue equivalents of the proteins, instead of the metastability reaction represented by Reaction 6. To demonstrate this procedure, let us write the formation reaction for the residue equivalent of CSG_METVO as

(8) $$\begin{array}{l} 4.656{\text{CO}}_{2(aq)}+1.935{\text{H}}_{2}\text{O}+1.166{\text{NH}}_{3(aq)}+0.020{\text{H}}_{2}{\text{S}}_{\left(aq\right)}\\ ⇌{\text{C}}_{4.656}{\text{H}}_{7.307}{\text{N}}_{1.166}{\text{O}}_{1.598}{\text{S}}_{0.020_{\left(\text{residue},\text{CSG\_METVO},aq\right)}}^{-0.101}+4.825{\text{O}}_{2(g)}+0.101{\text{H}}^{+} \end{array}$$

and that for the residue equivalent of CSG_METJA as

(9) $$\begin{array}{l} 4.821{\text{CO}}_{2(aq)}+1.966{\text{H}}_{2}\text{O}+1.208{\text{NH}}_{3(aq)}+0.026{\text{H}}_{2}{\text{S}}_{\left(aq\right)}\\ ⇌{\text{C}}_{4.821}{\text{H}}_{7.502}{\text{N}}_{1.208}{\text{O}}_{1.632}{\text{S}}_{0.026_{\left(\text{residue},\text{CSG\_METJA},aq\right)}}^{-0.105}+4.988{\text{O}}_{2(g)}+0.105{\text{H}}^{+}. \end{array}$$

Specific statements of Eqn. (M8) for Reactions 8 and 9 are, respectively,

(10) $$\begin{array}{c} A_{8}/2.303RT=\log K_{8}-\log\left(a_{{\text{C}}_{4.656}{\text{H}}_{7.307}{\text{N}}_{1.166}{\text{O}}_{1.598}{\text{S}}_{0.020_{\left(\text{residue},\text{CSG\_METVO},aq\right)}}^{-0.101}}f_{{\text{O}}_{2(g)}}^{4.825}a_{\text{H}+}^{0.101}\right)\\ +\log\left(a_{{\text{CO}}_{2(aq)}}^{4.656}a_{{\text{H}}_{2}\text{O}}^{1.935}a_{{\text{NH}}_{3(aq)}}^{1.166}a_{{\text{H}}_{2}\text{S}}^{0.020}\right) \end{array}$$

and

(11) $$\begin{array}{c} A_{9}/2.303\text{RT}=\text{log}K_{9}-\text{log}\left(a_{C_{4.821}H_{7.502}N_{1.208}O_{1.632}S_{0.026_{\left(\text{residue},\text{CSG}\_\text{METJA},\text{aq}\right)}}^{-0.105}}f_{O_{2(g)}}^{4.988}a_{H+}^{0.105}\right)\\ +\text{log}\left(a_{CO_{2(\mathrm{aq})}}^{4.821}a_{H_{2}O}^{1.966}a_{NH_{3(\mathrm{aq})}}^{1.208}a_{H_{2}S}^{0.026}\right). \end{array}$$

At metastable equilibrium, ***A***_8 _= ***A***_9_, *i.e*. the chemical affinities of the formation reactions of the residue equivalents are equal. Values of log *K*_8 _= -367.714 and log *K*_9 _= -379.687 can be obtained using standard molal Gibbs energies at 25°C and 1 bar of the basis species and of the ionized proteins at pH 7 (see Fig. 4b). Let us also substitute the reference activities of the basis species described in the Methods and log $f_{{\text{O}}_{2(g)}}$ = -80 to write

(12) $$A_{8}/2.303RT=0.189-\log a_{{\text{C}}_{4.656}{\text{H}}_{7.307}{\text{N}}_{1.166}{\text{O}}_{1.598}{\text{S}}_{0.020_{\left(\text{residue},\text{CSG\_METVO},aq\right)}}^{-0.101}}$$

and

(13) $$A_{9}/2.303RT=0.593-\log a_{{\text{C}}_{4.821}{\text{H}}_{7.502}{\text{N}}_{1.208}{\text{O}}_{1.632}{\text{S}}_{0.026_{\left(\text{residue},\text{CSG\_METJA},aq\right)}}^{-0.105}}.$$

There are three unknowns in Eqns. (12) and (13). Conservation of protein length leads to a third equation:

(14) $$a_{{\text{C}}_{4.656}{\text{H}}_{7.307}{\text{N}}_{1.166}{\text{O}}_{1.598}{\text{S}}_{0.020_{\left(\text{residue},\text{CSG\_METVO},aq\right)}}^{-0.101}}+a_{{\text{C}}_{4.821}{\text{H}}_{7.502}{\text{N}}_{1.208}{\text{O}}_{1.632}{\text{S}}_{0.026_{\left(\text{residue},\text{CSG\_METJA},aq\right)}}^{-0.105}}=1.083,$$

where the value on the right-hand side corresponds to initial activities of the proteins each equal to 10^-3^. Solving Eqns. (12)–(14) gives $a_{{\text{C}}_{4.656}{\text{H}}_{7.307}{\text{N}}_{1.166}{\text{O}}_{1.598}{\text{S}}_{0.020_{\left(\text{residue},\text{CSG\_METVO},aq\right)}}^{-0.101}}$ = 0.307, $a_{{\text{C}}_{4.821}{\text{H}}_{7.502}{\text{N}}_{1.208}{\text{O}}_{1.632}{\text{S}}_{0.026_{\left(\text{residue},\text{CSG\_METJA},aq\right)}}^{-0.105}}$ = 0.776 and ***A***_(8 or 9)_/2.303*RT *= 0.703.

The addition of any protein to the system increases by one the number of unknowns in Eqn. (14) but also provides another equation in the form of Eqns. (12) and (13). The procedure to set up and solve these equations has been encoded in a general form in CHNOSZ and was used to produce the diagrams shown in Fig. 5. The CHNOSZ program includes options to analyze the protein formation reactions using whole protein formulas or their residue equivalents, which were used to construct Figs. 5a and 5b, respectively. The logarithm of total activity of protein residues is 0.8211 in each of these figures, which corresponds to the sum of the activities of the residue equivalents of the ten model proteins whose starting activities are 10^-3^.

Another way of representing the chemical speciation in a protein system is on a degree of formation diagram. The degree of formation of the *k*th protein (α_*k*_) can be calculated from

(15) $$\alpha_{k}=a_{\text{residue},k}/\sum_{j=1}^{\hat{j}}a_{\text{residue},j},$$

where $\hat{j}=\hat{k}$ denotes the number of proteins in the system, $\sum_{1}^{\hat{j}}a_{\text{residue},j}$ represents the total activity of protein residues, and $\sum_{1}^{\hat{k}}\alpha_{k}=1$. The degrees of formation of the proteins corresponding to the logarithms of activities shown in Fig. 5b are depicted in the figure in Additional File 2. This degree of formation diagram aids in visualization of the computed relative abundances of the proteins on a non-logarithmic scale.

The residue-equivalent approach was used in this study only to produce the diagrams shown in Fig. 5b and Additional File 2. The predominance diagrams shown elsewhere were produced using whole protein formulas in the formation reactions. Extending the residue-equivalent method to these diagrams would subtly alter the positions of the predominance field boundaries, more so for reactions between proteins that differ significantly in length. The differences in the locations of the predominance field boundaries can be assessed in part by comparing the locations of the crossover between predominant proteins in Figs. 5a and 5b.

### Temperature and pressure diagrams

**Figure 6:**
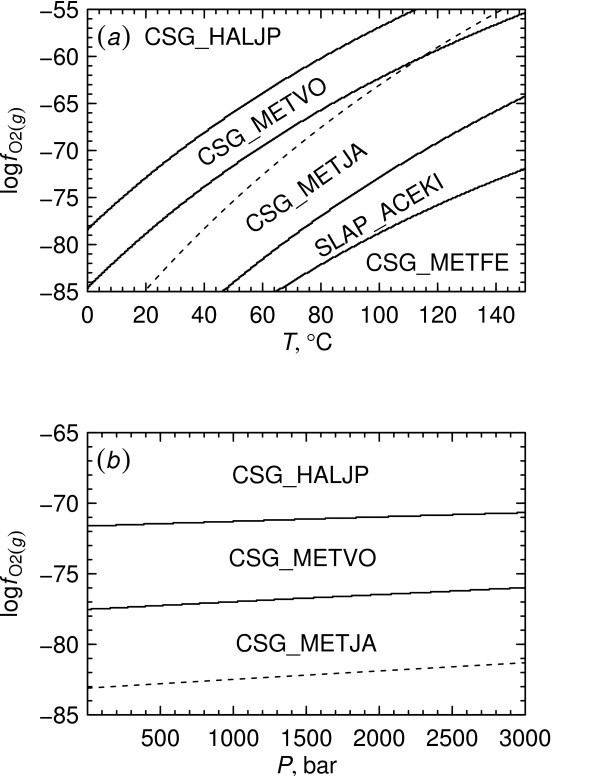
**Temperature and pressure dependence of protein metastability**. Predominance diagrams were constructed as a function of log $f_{{\text{O}}_{2(g)}}$ and (*a*) temperature at *P*_SAT _or (*b*) pressure at 25°C. The dashed line in each diagram represents the lower stability limit of H_2_O.

The approach described above for constructing Fig. 3 in CHNOSZ was used to produce the diagrams shown in Figs. 6a and 6b. These diagrams portray the metastabilities among the predominant model proteins as a function of temperature or pressure and log $f_{{\text{O}}_{2(g)}}$. It is immediately apparent that log $f_{{\text{O}}_{2(g)}}$ and temperature have a close relationship along a reaction boundary. For all of the reactions represented by the predominance field boundaries in Fig. 6a, increasing temperature is accompanied by an increase in the value of log $f_{{\text{O}}_{2(g)}}$. Hence, for a small positive increment in temperature at constant log $f_{{\text{O}}_{2(g)}}$, the metastability of CSG_METJA increases relative to that of CSG_METVO. If values of log $f_{{\text{O}}_{2(g)}}$ instead correspond as a function of temperature to the water stability limit (shown by the dashed line in Fig. 6a), increasing temperature would actually favor the formation of CSG_METVO relative to CSG_METJA.

We can recover nominal values of log $f_{{\text{O}}_{2(g)}}$ in the natural environments of *M. voltae *and *M. jannaschii *from geochemical data. The first of these organisms was originally isolated from the sediment of an estuary [42] and the other inhabits submarine hydrothermal vent environments [43]. Values of $a_{{\text{H}}_{2(aq)}}$ (activity of dissolved hydrogen) were taken from [44] and converted to log $f_{{\text{O}}_{2(g)}}$ using the law of mass action for H_2_O ⇌ H_2(*aq*) _+ 0.5O_2(*g*) _evaluated at 25°C and 1 bar to calculate a nominal range of log $f_{{\text{O}}_{2(g)}}$ for estuarine sediment of -73 to -70. Values of log $f_{{\text{O}}_{2(g)}}$ obtaining in mixed hydrothermal vent fluid and seawater at 100°C are in the range of -65 to -60 [45]. The first of these ranges would plot near the CSG_HALJP – CSG_METVO boundary in Fig. 6a at 25°C and the second one near the boundary between CSG_METVO and CSG_METJA at 100°C. This observation might support the notion that proteins from hyperthermophilic organisms like *M. jannaschii *are thermodynamically favored relative to those from mesophilic organisms by increasing temperature accompanied by changes in the geochemical oxidation state.

It appears in Fig. 6b that increasing pressure also generally favors those proteins in lower oxidation states, but that the dependence of equilibrium log $f_{{\text{O}}_{2(g)}}$ values on pressure is small relative to their dependence on temperature.

### Proteins as chemical activity buffers

The chemical activities of basis species buffered by reacting protein assemblages correspond to the locations of the (pseudo)invariant points on metastable equilibrium predominance diagrams. Equal activities of three proteins correspond to the triple point, which is a pseudoinvariant point, in the predominance diagram shown in Fig. 3b. The number of independent variables on the axes of this diagram is two; in an eight-dimensional predominance diagram (of temperature, pressure and six chemical activities) one could distinguish the true invariant points in this system where nine proteins coexist with equal metastable equilibrium activities.

Let us ask what are the activities of CO_2(*aq*) _, H_2_O, NH_3(*aq*) _and H_2_S_(*aq*) _if they are buffered by a hypothetical metastable assemblage made up of the proteins from the METXX organisms listed in Table 1, at *T *= 100°C, *P *= 1000 bar, pH 7, log $f_{{\text{O}}_{2(g)}}$ = -58 and activities of proteins equal to 10^-3^. At this temperature, pressure and pH the calculated charge of the cell-surface protein from *M. jannaschii *is -64.933. Consider then the formation reaction for this protein, which except for charge is equal to Reaction 4:

(16) $$\begin{array}{l} 2555{\text{CO}}_{2(aq)}+1042{\text{H}}_{2}\text{O}+640{\text{NH}}_{3(aq)}+14{\text{H}}_{2}{\text{S}}_{\left(aq\right)}\\ ⇌{\text{C}}_{2555}{\text{H}}_{3967.067}{\text{N}}_{640}{\text{O}}_{865}{\text{S}}_{14_{\left(\text{CSG\_METJA},aq\right)}}^{-64.933}+2643.5{\text{O}}_{2(g)}+64.933{\text{H}}^{+} \end{array}$$

A rearranged statement of Eqn. (M8) for this reaction can be written as

(17) $$\begin{array}{c} 530{\bar{A}}_{16}/2.303RT-2555\log a_{{\text{CO}}_{2(aq)}}-640\log a_{{\text{NH}}_{3(aq)}}-14\log a_{{\text{H}}_{2}{\text{S}}_{\left(aq\right)}}-1042\log a_{{\text{H}}_{2}\text{O}}\\ =\log K_{16}-\log a_{{\text{C}}_{2494}{\text{H}}_{3967.067}{\text{N}}_{613}{\text{O}}_{841}{\text{S}}_{9(\text{CSG\_METJA},aq)}^{-64.933}}-2463.5\log f_{{\text{O}}_{2(g)}}-64.933\log a_{\text{H}+} \end{array}$$

where $\bar{A}$_16 _≡ ***A***_16_/530, and the right-hand side works out to -4772.316 at the conditions stated above. At metastable equilibrium, the values of $\bar{A}$_16 _and its counterparts for the other proteins in the hypothetical assemblage are all equal. It follows that we can combine Eqn. (17) with its counterparts for the four other proteins, dropping the subscripts on $\bar{A}$, to write

(18) $$\left[\begin{array}{ccccc} 530 & 2555 & 1042 & 640 & 14\\ 571 & 2812 & 1066 & 747 & 16\\ 553 & 2575 & 1070 & 645 & 11\\ 278 & 1362 & 519 & 355 & 4\\ 571 & 2815 & 1071 & 747 & 14 \end{array}\right]\times \left[\begin{array}{c} \bar{A}/2.303RT\\ -\log a_{{\text{CO}}_{2(aq)}}\\ -\log a_{{\text{H}}_{2}\text{O}}\\ -\log a_{{\text{NH}}_{3(aq)}}\\ -\log a_{{\text{H}}_{2}S_{\left(aq\right)}} \end{array}\right]=\left[\begin{array}{c} -4772.316\\ -5785.204\\ -5021.307\\ -2683.266\\ -5825.691 \end{array}\right],$$

where the rows on the right-hand side and in the stoichiometric matrix on the left-hand side correspond to the proteins from the METXX organisms listed in Table 1. Solving Eqn. (18) gives $\bar{A}$/2.303*RT *= -0.739, log $a_{{\text{CO}}_{2(aq)}}$ = -8.44, log $a_{{\text{H}}_{2}\text{O}}$ = 7.92, log $a_{{\text{NH}}_{3(aq)}}$ = 27.92 and log $a_{{\text{H}}_{2}{\text{S}}_{\left(aq\right)}}$ = -13.09. These values signify that the formation reactions of the proteins per residue are energetically unfavorable ($\bar{A}$ is negative) and that the hypothetical protein assemblage may not be metastably present (for example, the large positive values for $a_{{\text{H}}_{2}\text{O}}$ and $a_{{\text{NH}}_{3(aq)}}$ differ from probable natural ranges). Unambiguous identification of a natural metastable protein assemblage may require more comprehensive calculations coupled with insight gained from experiments and observations in the field.

**Figure 7:**
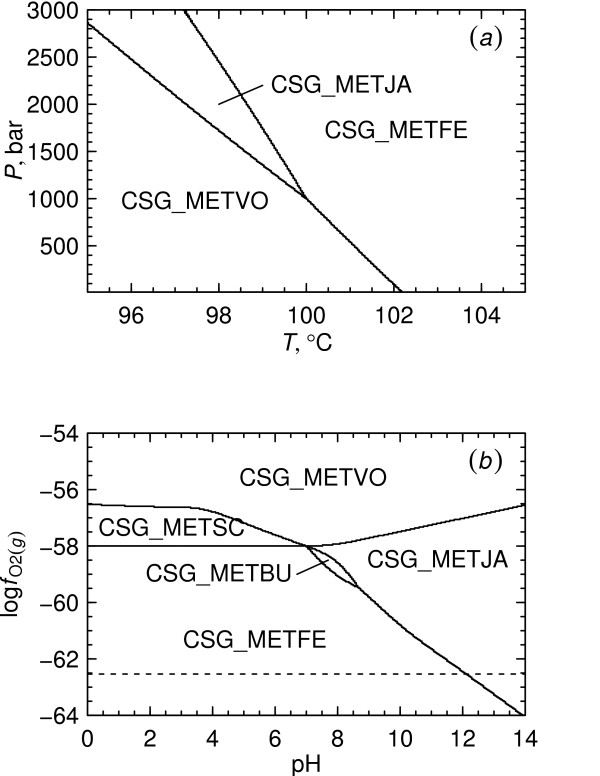
**Metastabilities of proteins around a pseudoinvariant point**. Activities of CO_2(*aq*)_, NH_3(*aq*)_, H_2_S_(*aq*) _and H_2_O were calculated using a buffer consisting of the proteins from the METXX organisms listed in Table 1. The variables set in the buffer calculation were activities of proteins equal to 10^-3^, *T *= 100°C, *P *= 1000 bar, log $f_{{\text{O}}_{2(g)}}$ = -58 and pH 7. The diagrams show the variation of protein metastability as a function of (*a*) temperature and pressure and (*b*) log $f_{{\text{O}}_{2(g)}}$ and pH. The dashed line in (*b*) represents the lower stability limit of H_2_O at this temperature and pressure.

The pseudoinvariant point representing the buffer assemblage described above is shown in Figs. 7a and 7b. The same pseudoinvariant point is present in both figures, but different variables are projected onto each diagram. The temperature-pressure relationships appearing in Fig. 7a suggest that metastability of CSG_METJA increases relative to that of CSG_METVO with increasing temperature and/or pressure, but that the sensitivity to temperature is much greater than that to pressure. These relationships are also apparent in Figs. 6a and 6b. In the projection of Fig. 7a all the proteins at the pseudoinvariant point are not visible, but in Fig. 7b convergence of the five predominance fields is apparent. Note the similarity in Figs. 7b and 3a of the reaction boundary between CSG_METVO and CSG_METJA, as well as the nearly horizontal boundary between CSG_METSC and CSG_METFE, which would be expected from the closeness of their ionization states as a function of pH (see Fig. 4a for the ionization states at 25°C).

### Concluding remarks

A computer program called CHNOSZ was introduced in this paper for producing metastable equilibrium chemical activity diagrams for proteins. The methods used here were borrowed from geochemistry, and the program with the accompanying thermodynamic database is suitable for performing thermodynamic calculations in inorganic and mineral systems as well as organic and biochemical systems, or combinations thereof.

To investigate the utility of the program for a geochemical description of protein reactions, metastability diagrams were produced for surface-layer proteins from a number of bacteria and archaea. The diagrams show either the metastably predominant proteins as a function of two intensive variables or the metastable equilibrium chemical activities of proteins as a function of one variable. The primary variables of interest in this study were log $f_{{\text{O}}_{2(g)}}$, pH, temperature and pressure. It was found that the predicted metastable equilibrium state of the system responded dramatically to changes in these variables. Representing the proteins in reactions by their residue equivalents instead of whole protein formulas gave rise to predicted equilibrium states in which many proteins coexist metastably with comparable chemical activities.

In the preceding sections we have considered the theoretical metastable equilibrium relationships among only a few model proteins. Because the software is now available to do so, a plethora of predictions concerning the energetically favorable outcomes of any number of overall protein mutation reactions is now within reach. Consideration of the results presented above, and of the wide range of model systems that could potentially be investigated in a similar manner, leads to the conclusion that the metastable equilibrium distribution of proteins in many cases does not mirror geobiochemical reality. Nevertheless, the ability to quantify the characteristics of metastable equilibrium reference states as a function of geochemical variables may be of utility in identifying specific pathways in evolution where the resulting proteins are relatively energetically favored. These particular outcomes may reflect a tendency for natural selection to increase the fit between phenotypes and their environments [46].

A thermodynamic and geochemical perspective on the relative metastabilities of proteins permits a quantitative integration of observations on the geosphere and biosphere. This study has only touched the surface of the myriad possible environments and organisms, the properties and chemical compositions of which are becoming more well constrained through experiment and observation. As these data grow in abundance, they will provide other opportunities where thermodynamic description of the chemical speciation of proteins can be tested and calibrated.

## Methods

The thermodynamic conventions and relations used to compute the relative metastabilities of proteins in the present study are summarized below. The computational assessment depends first on the adoption of standard states for the species appearing in chemical reactions.

### Standard state conventions

The standard state convention adopted for aqueous species other than H_2_O corresponds to unit activity of a hypothetical one molal solution referenced to infinite dilution at any temperature and pressure [30,47]. The conventional standard molal thermodynamic properties of both the aqueous electron and proton are taken to be zero at all temperatures and pressures [48]. For gases, the standard state convention is unit fugacity of the hypothetical pure ideal gas at 1 bar and any temperature. The standard state convention adopted for solids and liquids, including H_2_O, corresponds to unit activity of the pure substance at any temperature and pressure.

### Protein formation and metastability reactions

The compositions of species of interest, such as proteins, are represented by linear combination of the compositions of basis species in a system (for an application in geochemical systems, see Ref. [49]). The number of basis species is the minimum required to write formation reactions for all possible species of interest. There are no thermodynamic restrictions on the actual identities of the basis species, and the basis species do not necessarily correspond to thermodynamic components in the system of interest [50]. Hence, the choice of basis species may be constrained by the chemical activities that can be measured in a system or that are thought to behave as perfectly mobile components [22]. The basis species used in the present study are CO_2(*aq*)_, H_2_O, NH_3(*aq*)_, H_2_S_(*aq*)_, H^+ ^and O_2(*g*)_.

Let a generic chemical formula for the *j*th ionized protein be written as ${\text{C}}_{C_{j}}{\text{H}}_{H_{j}}{\text{N}}_{N_{j}}{\text{O}}_{O_{j}}{\text{S}}_{S_{j}}^{Z_{j}}$, where *C*_*j*_, *H*_*j*_, *N*_*j*_, *O*_*j*_, *S*_*j *_and *Z*_*j *_denote the number of moles of the corresponding element (or charge) in one mole of protein. These coefficients can be non-integer and positive or negative (*e.g*., *Z*_*j *_usually is negative at some alkaline pHs). The formation reaction from basis species of one mole of the *j*th protein can be written as

(M1) $$\begin{array}{l} C_{j}{\text{CO}}_{2(aq)}+N_{j}{\text{NH}}_{3(aq)}+S_{j}{\text{H}}_{2}{\text{S}}_{\left(aq\right)}+Z_{j}{\text{H}}^{+}+\left(\frac{H_{j}-3N_{j}-2S_{j}-Z_{j}}{2}\right){\text{H}}_{2}\text{O}\\ +\left(\frac{O_{j}-2C_{j}-\left(\frac{H_{j}-3N_{j}-2S_{j}-Z_{j}}{2}\right)}{2}\right){\text{O}}_{2(g)}⇌{\text{C}}_{C_{j}}{\text{H}}_{H_{j}}{\text{N}}_{N_{j}}{\text{O}}_{O_{j}}{\text{S}}_{S_{j}}^{Z_{j}}. \end{array}$$

The reaction coefficients on the basis species in Reaction M1 are completely determined by the chemical formulas of the protein and of the basis species. Depending on the sign of the coefficients in front of the basis species, they would appear in specific statements of Reaction M1 as reactants or products.

A generic metastability reaction between two proteins (*j *= 1 and *j *= 2) can be written as

(M2) $$\begin{array}{c} \frac{1}{n_{1}}{\text{C}}_{C_{1}}{\text{H}}_{H_{1}}{\text{N}}_{N_{1}}{\text{O}}_{O_{1}}{\text{S}}_{S_{1}}^{Z_{1}}+\left(\frac{C_{2}}{n_{2}}-\frac{C_{1}}{n_{1}}\right){\text{CO}}_{2(aq)}+\left(\frac{N_{2}}{n_{2}}-\frac{N_{1}}{n_{1}}\right){\text{NH}}_{3(aq)}+\left(\frac{S_{2}}{n_{2}}-\frac{S_{1}}{n_{1}}\right){\text{H}}_{2}{\text{S}}_{\left(aq\right)}\\ \begin{array}{c} +\left(\frac{Z_{2}}{n_{2}}-\frac{Z_{1}}{n_{1}}\right){\text{H}}^{+}+\left(\frac{\left(\frac{H_{2}}{n_{2}}-\frac{H_{1}}{n_{1}}\right)-3\left(\frac{N_{2}}{n_{2}}-\frac{N_{1}}{n_{1}}\right)-2\left(\frac{S_{2}}{n_{2}}-\frac{S_{1}}{n_{1}}\right)-\left(\frac{Z_{2}}{n_{2}}-\frac{Z_{1}}{n_{1}}\right)}{2}\right){\text{H}}_{2}\text{O}\\ +\left(\frac{\left(\frac{O_{2}}{n_{2}}-\frac{O_{1}}{n_{1}}\right)-2\left(\frac{C_{2}}{n_{2}}-\frac{C_{1}}{n_{1}}\right)-\left(\frac{\left(\frac{H_{2}}{n_{2}}-\frac{H_{1}}{n_{1}}\right)-3\left(\frac{N_{2}}{n_{2}}-\frac{N_{1}}{n_{1}}\right)-2\left(\frac{S_{2}}{n_{2}}-\frac{S_{1}}{n_{1}}\right)-\left(\frac{Z_{2}}{n_{2}}-\frac{Z_{1}}{n_{1}}\right)}{2}\right)}{2}\right){\text{O}}_{2(g)}\\ ⇌\frac{1}{n_{2}}{\text{C}}_{C_{2}}{\text{H}}_{H_{2}}{\text{N}}_{N_{2}}{\text{O}}_{O_{2}}{\text{S}}_{S_{2}}^{Z_{2}}, \end{array} \end{array}$$

which corresponds to the difference between specific statements of Reaction M1 for *j *= 2 and *j *= 1, divided by *n*_2 _or *n*_1_, respectively. Here, 1/*n*_1 _and 1/*n*_2 _denote the conservation coefficients for the corresponding proteins. Reaction M2 is balanced with respect to mass and charge for any values of *n*_1 _and *n*_2_. If *n*_1 _= *n*_2 _= 1, Reaction M2 denotes the mass balance constraints for the formation of one mole of product protein at the expense of one mole of reactant protein. Other values may be chosen for *n*_1 _and *n*_2_, depending on what is specified about the conservation constraints in the system. For example, if *n*_1 _= *C*_1 _and *n*_2 _= *C*_2_, the protein metastability reaction conserves carbon [18] (*i.e*., the coefficient on CO_2(*aq*) _in Reaction M2 becomes zero). The protein metastability reactions considered in the present study are written for *n*_*j *_equal to the length of the *j*th protein.

### Relation of reaction energetics to activities of basis species

The standard Gibbs energy of the *r*th formation or metastability reaction ($\Delta G_{r}^{\circ }$) can be expressed as

(M3) $$\Delta G_{r}^{\circ }=\sum_{r}{\hat{n}}_{i,r}\Delta G_{r}^{\circ },$$

where ${\hat{n}}_{i,r}$ and $\Delta G_{i}^{\circ }$ denote, respectively, the stoichiometric reaction coefficient and standard molal Gibbs energy of formation from the elements of the *i*th basis species or protein in the reaction. For products in a reaction, ${\hat{n}}_{i,r}$ > 0. The corresponding equilibrium constant of the reaction (*K*_*r*_) is given by

(M4) $$\log K_{r}=-\Delta G_{r}^{\circ }/2.303RT.$$

The equilibrium constant, like $\Delta G_{r}^{\circ }$, is a standard-state property which is is independent of composition and depends on temperature and pressure. The non-standard-state counterpart to *K*_*r *_is the activity product of the reaction (*Q*_*r*_), which can be computed using

(M5) $$Q_{r}\equiv \prod_{i}a_{i}^{{\hat{n}}_{i,r}},$$

where *a*_*i *_represents the chemical activity of the *i*th species in the reaction. For gaseous species, *a*_*i *_in Eqn. (M5) is replaced by the fugacity of the species (*f*_*i*_). Activity and fugacity coefficients are taken in a first approximation in this study to be unity.

The activity or fugacity of the *i*th aqueous or gaseous component is related to its chemical potential (*μ*_*i*_) by [6]

(M6) $$\mu_{i}=\mu_{i}^{\circ }+RT\ln\frac{f_{i}}{f_{i}^{\circ }}=\mu_{i}^{\circ }+RT\ln a_{i},$$

where $\mu_{i}^{\circ }$ denotes the standard chemical potential of the *i*th species and $f_{i}^{\circ }$ stands for the fugacity of the species in its standard state, which is unity for gases.

The chemical affinities of reactions (***A***_*r*_) can be computed from [51]

(M7) ***A***_*r *_= 2.303*RT *log (*K*_*r*_/*Q*_*r*_),

which can be combined with Eqn. (M5) to write for Reaction M2

(M8) $$\log K_{r}=A_{r}/2.303RT+\log\frac{a_{{\text{C}}_{C_{2}}{\text{H}}_{H_{2}}{\text{N}}_{N_{2}}{\text{O}}_{O_{2}}{\text{S}}_{S_{2}}^{Z_{2}}}^{1/n_{2}}}{a_{{\text{C}}_{C_{1}}{\text{H}}_{H_{1}}{\text{N}}_{N_{1}}{\text{O}}_{O_{1}}{\text{S}}_{S_{1}}^{Z_{1}}}^{1/n_{1}}}+\log\left(a_{{\text{CO}}_{2(aq)}}^{{\hat{n}}_{{\text{CO}}_{2}}}a_{{\text{H}}_{2}\text{O}}^{{\hat{n}}_{{\text{H}}_{2}\text{O}}}a_{{\text{NH}}_{3(aq)}}^{{\hat{n}}_{{\text{NH}}_{3}}}f_{{\text{O}}_{2(aq)}}^{{\hat{n}}_{{\text{O}}_{2}}}a_{{\text{H}}_{2}{\text{S}}_{\left(aq\right)}}^{{\hat{n}}_{{\text{H}}_{\text{2}}\text{S}}}a_{\text{H}+}^{{\hat{n}}_{\text{H}+}}\right).$$

In an equilibrium state, ***A***_*r *_= 0 for metastability reactions and Eqn. (M8) reduces to the logarithmic analog of the law of mass action equation for Reaction M2.

### Reference activities of basis species and proteins

The reference temperature and pressure correspond to 25°C and 1 bar, respectively. The reference chemical activities of basis species used in this study are given by log $a_{{\text{H}}_{2}\text{O}}$ = 0, log $a_{{\text{CO}}_{2(aq)}}$ = -3, log $a_{{\text{NH}}_{3(aq)}}$ = -4, log $a_{{\text{H}}_{2}{\text{S}}_{\left(aq\right)}}$ = -7 and log *a*_H+ _= -7 (pH 7). The reference value for log $a_{{\text{H}}_{2}\text{O}}$ corresponds to pure water, and the others are nominal values that generally fall within the compositional ranges of hydrothermal fluids and seawater [52]. The reference chemical activities of proteins are taken to be 10^-3^, which is a nominal value that is similar to experimental concentrations used in protein unfolding studies [53].

### Equations of state

The standard molal thermodynamic properties of aqueous species as a function of temperature and pressure can be evaluated using the revised Helgeson-Kirkham-Flowers (HKF) equations of state [30-33,54,55]. The temperature dependence of the standard molal thermodynamic properties of crystalline, gaseous and liquid species other than H_2_O are calculated using a standard equation for heat capacity [34,35,56]. For the basis species other than H^+ ^and *e*^-^, values of the standard molal thermodynamic properties and of the equations of state parameters were taken from Refs. [55,57] (CO_2(*aq*)_, NH_3(*aq*) _and H_2_S_(*aq*)_) and [58,59] (O_2(*g*)_). The equations of state adopted for liquid H_2_O in the present study are those used in the SUPCRT92 software package [24].

### Group additivity algorithms for ionized proteins

The standard molal properties and revised HKF equations of state parameters of ionized proteins are calculated in the present study using group additivity algorithms and data taken from Ref. [18] and outlined briefly below. The standard molal Gibbs energy of the *j*th unfolded protein with net charge denoted by *Z*_*j *_($\Delta G_{UP_{j}^{Z_{j}}}^{\circ }$) can be written as

(M9) $$\Delta G_{UP_{j}^{Z_{j}}}^{\circ }=\Delta G_{UP_{j}^{0}}^{\circ }+\Delta G_{\text{ion},j}^{\circ },$$

where $\Delta G_{UP_{j}^{0}}^{\circ }$ stands for the standard molal Gibbs energy of the completely neutral (nonionized) unfolded protein and $\Delta G_{\text{ion},j}^{\circ }$ stands for the contribution of ionization of sidechain and terminal groups to the standard molal Gibbs energy of the ionized protein. The latter term can be calculated by first writing

(M10) $$\Delta G_{\text{ion},j}^{\circ }=\sum_{i}n_{i,j}\alpha_{i}\Delta G_{\text{ion},i}^{\circ },$$

where, for the *i*th type of ionizable sidechain or backbone group, *n*_*i*, *j *_represents the number of moles of the group in one mole of protein, *α*_*i *_denotes the degree of ionization of the group (0 <*α*_*i *_< 1), and $\Delta G_{\text{ion},i}^{\circ }$ corresponds to the standard molal Gibbs energy of ionization of the group. Values of *α*_*i *_and $\Delta G_{\text{ion},j}^{\circ }$ in Eqn. (M10) were taken in a simple approximation to be equal for all occurrences of a given ionizable group. It may be possible to refine this approach in the future by taking account of interactions of charged residues on the protein surfaces (Ref. [60] and others since).

Although  and $\Delta G_{\text{ion},j}^{\circ }$ in Eqns. (M9) and (M10) are functions only of temperature and pressure for any protein in a defined charge state, *α*_*i *_in Eqn. (M10) is a function of temperature, pressure, and solution pH [18]. Hence, $\Delta G_{\text{ion},j}^{\circ }$ and  can be effectively computed for a given protein in different ionization states as a function of pH as well as of temperature and pressure. The net charge of the *j*th protein (*Z*_*j*_) as a function of temperature, pressure and pH can be calculated using

(M11) $$Z_{j}=\sum_{i}n_{i,j}\alpha_{i}Z_{i},$$

where *Z*_*i *_denotes the charge (+1 or -1) of the *i*th ionized group and *α*_*i *_(also in Eqn. M10) is given by

(M12) $$\alpha_{i}=\frac{1}{1+10^{Z_{i}(\text{pH}-\text{p}K_{i})}},$$

where p*K*_*i *_represents the negative logarithm of the equilibrium constant for the deprotonation reaction of the *i*th ionizable group.

For a protein composed of a single polypeptide chain, the values of $\Delta G_{UP_{j}^{0}}^{\circ }$ in Eqn. (M9) can be calculated from the group additivity algorithm represented by [17,18]

(M13) $$\Delta G_{UP_{j}^{0}}^{\circ }=\Delta G_{\left[\text{AABB}\right]}^{\circ }+(n_{j}-1)\Delta G_{\left[\text{UPBB}\right]}^{\circ }+\sum_{i}^{20}n_{{\left[SC\right]}_{i}}\Delta G_{{\left[SC\right]}_{i}}^{\circ },$$

where $\Delta G_{\left[\text{AABB}\right]}^{\circ },\Delta G_{\left[\text{UPBB}\right]}^{\circ }$ and $\Delta G_{{\left[SC\right]}_{i}}^{\circ }$ denote the standard molal Gibbs energies of the amino acid backbone group, unfolded protein backbone group, and the *i*th type of amino acid sidechain group, respectively, $n_{{\left[SC\right]}_{i}}$ stands for the number of moles of the *i*th type of amino acid sidechain group in one mole of the protein, and

(M14) $$n_{j}=\sum_{i=1}^{20}n_{{\left[SC\right]}_{i}}$$

represents the total number of amino acid residues, or length of the protein. Values of $n_{{\left[SC\right]}_{i}}$ for the model proteins considered in the present study were retrieved from the Swiss-Prot/UniProt protein sequence database [37] (see Table 1).

The thermodynamic properties of unfolded aqueous proteins calculated using the above equations are taken in a first approximation to be representative of the proteins of interest, which may be folded and/or present in crystalline form in cells. Two observations lend support to the applicability of the unfolded protein reference state for the present calculations: 1) The standard molal Gibbs energies of protein folding would tend to cancel each other in metastability reactions, in which proteins appear on both sides of the reaction. 2) The Gibbs energy of unfolding for a small to average-sized protein is about two or three orders of magnitude smaller than the standard molal Gibbs energy for the unfolded protein itself. For example, the Gibbs energy of unfolding of chicken lysozyme is ~14.5 kcal mol^-1 ^at 25°C [61], but the standard molal Gibbs energy of this protein at 25°C and 1 bar is ~-4.2 × 10^3 ^kcal mol^-1 ^(see Figs. 2a and 2b). The size of the unfolding property in this case is much smaller than the *ca*. ± 5% uncertainty ascribed to the group additivity algorithm [18]. It should be noted, however, that the compositional consequences of protein folding include changes in ionization state, and preferential surface exposure of charged residues [1], which would be manifested by changes in the reaction coefficients of basis species that might affect the outcome of metastability calculations to a greater extent than the differences in Gibbs free energy alone.

## Competing interests

The authors declare that they have no competing interests.

## Supplementary Material

Additional file 1 Program script for generating figures. This text file contains the program script used to make the diagrams in Figs. 3, 4, 5, 6, 7. Use the commands listed at the top of the file to generate one or all of the figures on screen or in postscript format. Click here for file

Additional file 2 Degree of formation diagram. This file contains the degree of formation diagram related to Fig. 5b (see text) together with the program script used to make the figure. This additional material is the source of the graphical abstract for this paper. Click here for file
