# Supplementary material for: Calculation of the relative metastabilities of proteins using the CHNOSZ software package
Source: Geochem Trans. 2008 Oct 3;9:10. doi: 10.1186/1467-4866-9-10 (PMC2654789; doi:10.1186/1467-4866-9-10)
Supplement: Additional file 2 — Degree of formation diagram. This file contains the degree of formation diagram related to Fig. 5b (see text) together with the program script used to make the figure. This additional material is the source of the graphical abstract for this paper. [file 1467-4866-9-10-S2.pdf]

Additional File 2

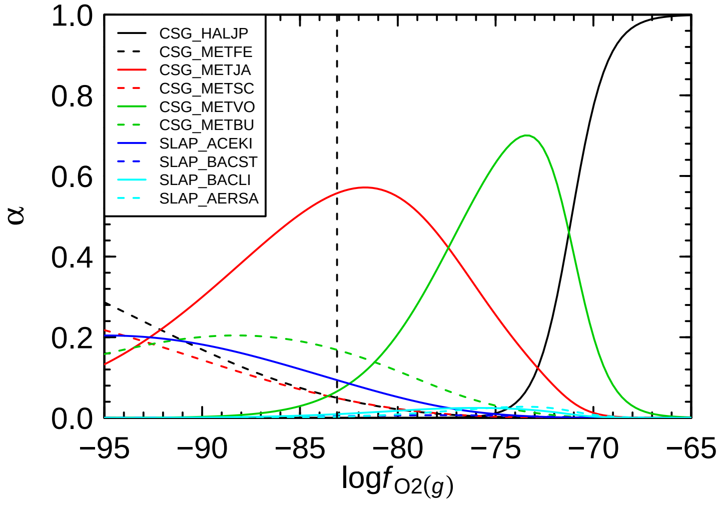

Calculated degrees of formation of ten surface-layer proteins as a function of $\log f_{O_{2(g)}}$ at 25 °C and 1 bar (see text for activities of basis species used). The vertical dashed line denotes the lower (reducing) stability limit of H_2O. The figure was created in CHNOSZ using the following commands:

```
# open plot file
thermo.postscript("alpha.ps")
# set sizes, margins, background color
par(cex=2,lwd=2,mar=c(3,3,1,1),bg="white")
# define basis species
basis("CHNOS+")
# load proteins
species(c("CSG_HALJP","CSG_METFE","CSG_METJA",
  "CSG_METSC","CSG_METVO","CSG_METBU",
  "SLAP_ACEKI","SLAP_BACST","SLAP_BACLI","SLAP_AERSA"))
# calculate affinities of formation reactions
a <- affinity(O2=c(-95,-65))
# plot metastability diagram with options for line
# type, color, and legend
diagram(a,residue=TRUE,alpha=TRUE,lty=rep(c(1,2),5),
  color=rep(palette()[1:5],each=2),
  legend.x="topleft",cex.names=0.5)
# plot water line
s <- subcrt(c("H2O","O2","H2"),c(-1,0.5,1),T=25)
abline(v=2*s$out$logK,lty=2)
# close plot file
dev.off()
```
